# Predictive factors for orchiectomy in adult’s spermatic cord torsion: a case‐control study

**DOI:** 10.1186/s12610-021-00122-y

**Published:** 2021-01-21

**Authors:** Fasnéwindé Aristide Kabore, Klovis Klifford Kabore, Moussa Kabore, Brahima Kirakoya, Clotaire Yameogo, Bienvenue Désiré Ky, Barnabé Zango

**Affiliations:** Department of Urology and Andrology, University Hospital Yalgado Ouedraogo of Ouagadougou, 03 BP7022 Ouaga 09, Ouagadougou, Burkina Faso

**Keywords:** Spermatic cord torsion, Orchiectomy, Orchidopexy, Predictive factors, Surgical emergency

## Abstract

**Background:**

Spermatic cord torsion (SCT) is a surgical emergency. Any delay in diagnosis or treatment may compromise the vital and functional prognosis of the testicle. The orchiectomy rate remains very high in the literature; it can even reach 50 % in certain series. Several factors have been reported in the literature as being significantly correlated with the risk of orchiectomy: duration of symptoms, the number of turns, the younger ages, etc. The objective of this study has been to analyse the predictive factors of orchiectomy in adult SCT in our context.

**Results:**

During the study period, 74 cases of SCT were included. Orchidopexy was performed in 43.2 % (32/74) versus orchiectomy in 56.8 % (42/74) of cases. The patients’ median age was 22 years (interquartile range (IQR) = 18–26.75 years). The duration of symptoms (*p* = 0.009), the previous medical management (*p* < 0.001), performing a scrotal ultrasonography (*p* = 0.004) were statistically significant at univariate analysis. On multivariate analysis only the previous medical management was statistically significant (*p* = 0.017).

**Conclusions:**

The orchiectomy rate was very high in our cohort. The previous medical management was the only significant predictive factor of orchiectomy on multivariate analysis. Our findings demonstrated that the delay in diagnosis is the determining factor in testicular loss in our context.

## Résumé

**Contexte:**

La torsion du cordon spermatique est une urgence chirurgicale. Tout retard dans le diagnostic ou le traitement peut compromettre le pronostic vital et fonctionnel du testicule. Le taux d'orchidectomie reste très élevé dans la littérature;il peut même atteindre 50 % dans certaines séries. Plusieurs facteurs ont été rapportés dans la littérature comme étant significativement corrélés au risque d'orchidectomie. L'objectif de cette étude était d'analyser les facteurs prédictifs d'orchidectomie dans la torsion du cordon spermatique de l'adulte dans notre contexte.

**Résultats:**

Durant la période d’étude 74 patients ont été inclus. Une orchidopexie a été réalisée dans 43,2 % (32/74) des cas et une orchidectomie dans 56,8 %(42/74) des cas. L'âge médian des patients était de 22 ans (écart interquartile=18-26,75 ans). La durée des symptômes (*p* = 0,009), la prise en charge médicale antérieure(*p*<0,001), la réalisation d'une échographie des bourses (*p* = 0,004) étaient significativement corrélées au risque d’orchidectomie à l’analyse univariée. A l'analyse multivariée, seule la prise en charge médicale antérieure était significativement corrélée au risque d’orchidectomie (*p*=0.017).

**Conclusion:**

Le taux d'orchidectomie était très élevé dans notre cohorte. La prise en charge médicale antérieure était le seul facteur prédictif significatif de l'orchidectomie à l'analyse multivariée. Nos résultats ont montré que le retard diagnostic est le facteur déterminant de la perte testiculaire dans notre contexte.

**Mots clés:**

Torsion du cordon spermatique, Orchidectomie, Orchidopexie, Facteurs prédictifs, Urgence chirurgicale

## Background

Spermatic cord torsion (SCT) is the rotation of the epididymotesticular block around its vasculo-deferential axis. This results in a strangulation of the vasculo-nerveous elements of the testicle. Its incidence is historically estimated by Williamson [1] at 1 per 4,000 men under 25. It constitutes a real diagnostic and therapeutic emergency. Indeed, any delay in diagnosis or treatment may compromise the vital and functional prognosis of the testicle. If the untwisting of testis is performed within 6 hours after the onset of the symptoms, a preservation of the testicle is possible in 90 % of the cases, compared to 10 % after 24 hours [2–4]. The orchiectomy rate remains very high in the literature; it can even reach 50 % in certain series [5–7]. This risk of testicular loss is what makes this condition so serious.

Several factors have been reported in the literature as being significantly correlated with the risk of orchiectomy in case of SCT: the consultation delay, the number of turns, the younger ages, etc. [8–10]. In Burkina Faso, Kaboré et al. [6] reported an orchiectomy rate of 55 % in a previous study and blamed the consultation delay for the high orchiectomy rate. However, one of the weaknesses of their study was related to the lack of testing for statistical comparisons. We hypothesize that there are other predictive factors of orchiectomy in SCT in our context. The aim of the present study was then to investigate these predictive factors of orchiectomy. To know these factors would improve the vital prognosis of the testicle by reducing the orchiectomy rate.

## Patients and methods

We carried out an unicentric case-control study over an 11-years (1st January 2005 to 31st December 2015). The study was conducted at the surgical emergency department of the Yalgado Ouédraogo Teaching Hospital of Ouagadougou (Burkina Faso). Patients meeting the following criteria were included: patients over 15 years of age, a surgical exploration that confirmed the SCT, a complete medical record.

During the study period, 80 patients were managed for SCT. Six patients have been excluded for incomplete data. The included patients (74) were divided into two groups: the case group and the control group. The case group was constituted by the patients who underwent an orchiectomy, and the control group by those who underwent orchidopexy.

The following parameters were considered for the purposes of this study: the patient’s age, the duration of symptoms prior to presentation (delay between the onset of the symptoms and the first consultation), the existence of previous medical management (whether the patient was seen in another care facility for the same or a similar complaint before the day of the surgery or not), the mode of hospital admission (patient referred from another hospital or not), the time to operating room (delay between the consultation in the emergency room and the surgery), performing an ultrasound (referred patient have performed or not an ultrasound before being referred), results of surgical exploration (orchiectomy vs. orchidopexy).

After surgical detorsion, if the testicle was immediately viable, an orchidopexy was performed. In case of doubtful viability of the testicle, it was placed in a saline bath for at least 10 minutes. If it was well recolored, an orchidopexy was then performed, otherwise an orchidectomy was performed.

### Statistical analysis

Age was categorized as either under or over 20 years old (based on the median age), duration of symptoms as either shorter or longer than 6 hours, time to operating room as either shorter or longer than 2 hours, and surgical outcome (orchiectomy or orchidopexy).

All statistical analyses were performed using R version 3.6.1. On univariate analysis, the Chi-Square test was used to measure the correlation between our variables of interest and the risk of orchiectomy. A p-value < 0.05 was considered to be significant.

Multivariate logistic regression was used to determine the significant orchiectomy predictive factors. Variables at or approaching significance on univariate analysis (p < 0.2) were entered in the models. All data has been anonymized.

## Results

The median age of the patients was 22 years (interquartile range (IQR) = 18–26.75 years) with values ranging from 15 years to 80 years old. Seventy-six percent (76 %) of the patients were referred from another health care facility. The median duration of symptoms was 24 hours (interquartile range (IQR) = 7.25–72 hours) with values ranging from 2 hours to 240 hours. Seventy-seven percent (77 %) of patients consulted more than six hours after the onset of symptoms. The median duration of symptoms in referred patients was 24 hours (interquartile range (IQR) = 9.25-96 hours) with values ranging from 2 hours to 240 hours. The median duration of symptoms in patients who consulted directly in our hospital was 24 hours (interquartile range (IQR) = 5.75–24 hours) with values ranging from 2 hours to 120 hours. The median duration of symptoms in patients who performed a scrotal ultrasound was 72 hours (interquartile range (IQR) = 24–120 hours) with values ranging from 2 hours to 240 hours. The median duration of symptoms in patients who did not perform scrotal ultrasound was 10 hours (interquartile range (IQR) = 4.5–24 hours) with values ranging from 2 hours to 96 hours. The orchidopexy rate was 43.2 % (32/74) and the orchiectomy rate was 56.8 % (42/74). Contralateral orchidopexy was performed in 38 cases during the same intervention.

On univariate analysis, the previous medical management, the duration of symptoms, the performance of a scrotal ultrasound were significantly different between the orchiectomy group and that of the orchidopexy (Table 1). The orchiectomy rate in referred patients was 62.5 % (35/56) versus 38.9 % (7/18) in those who consulted directly in our hospital. This difference was not statistically significant (*p* = 0.08).

**Table 1:** Patient characteristics, univariate analysis comparing testicular salvage (*n* = 74)

	Orchiectomy *n* = 42	Orchidopexy *n* = 32	*P* value
Age (years)			
[15-20]	19	13	0.69
[21-80]	23	19	
Previous medical management			
Yes	30	10	**<**0.001
No	12	22	
Mode of hospital admission			
Direct	07	11	0.08
Referred	35	21	
Duration of symptoms (hours)			
≤ 6	05	12	0.009
> 6	37	20	
Scrotal ultrasonography			
Yes	26	09	0.004
No	16	23	
Time to operating room (hours)			
≤ 2	12	15	0.06
> 2	29	07	

Multivariate analysis showed that the previous medical management was the only statistically significant orchiectomy predictors (Table 2).

**Table 2:** Multivariate analysis (*n* = 74)

	*P* value	Odds Ratio	95%CI
Previous medical management			
Yes	0.017	37.315	[1.52-38.64]
No			
Mode of hospital admission			
Direct	0.88	2.256	[0.18-8.26]
Referred			
Duration of symptoms (hours)			
≤ 6	0.581	0.709	[0.29-8.56]
> 6			
Scrotal ultrasonography			
Yes	0.12	1.662	[0.76-15.54]
No			
Time to operating room (hours)			
≤ 2	0.165	5.246	[0.65-15.37]
> 2			

*IC* Confidence Interval

## Discussion

We demonstrated in our cohort that the previous medical management was the only significant predictive factors of orchiectomy on multivariate analysis (*p* = 0.017). Our findings strengthen the conclusion of Bayne et al. [8] from the United States of America (USA). These authors reported that if the patient had previously been seen for the same symptomatology, this increased significantly the risk of orchiectomy. In developing countries, some missed diagnosis occurred in primary health-care centres. In these centres, there is sometimes a lack of qualified health agent who can recognize the signs of SCT. This situation results in diagnostic delay and therefore a delay in the patient’s transfer to a suitable health care facility.

The orchiectomy rate in patients who were referred vs. not referred was 62.5 % vs. 41.17 % respectively. However, that difference was not statistically significant (*p* = 0.08). Ramachandra et al. [11] in USA, did not find a significant correlation between the mode of hospital admission and the orchiectomy risk. Nevertheless, according to Ramachandra et al. [11], when the patient was referred, that increased the management delay, which was significantly related to the orchiectomy risk (p < 0.001). Therefore, the mode of hospital admission indirectly impacts the risk of orchiectomy by increasing the management delay. The Royal College of Surgeons in England recommends to perform surgical exploration in the hospital where the patient is seen for the first time [12]. The objective is to avoid transferring the patient in order to reduce the management delay. This approach is difficult to apply in our context because of a glaring lack of urologists and surgeons in these primary health care centres. Contrary to our cohort, the correlation between the surgical management delay and the risk of orchiectomy was significant in the series of Ramachandra et al. [11].

We found out a statistically significant correlation on univariate analysis between performing a scrotal ultrasound and the orchiectomy risk (*P* = 0.004). Indeed, performing a scrotal ultrasound may delay the patient’s transfer.

Zini et al. [13] showed that the delay between the patients’ arrival at the emergency department and the surgery was 2.6 times longer when an ultrasound was performed (p < 0.001). Preece et al. [14] made the same finding even though the correlation was not significant. Therefore, we agree with Sauvat et al. [15] and Zini et al. [13] that, if there is a slightest doubt, surgical exploration should immediately be carried out in order to reduce the management delay and hence the risk of orchiectomy. Surgical exploration remains the gold standard in the diagnosis of SCT [16]. This attitude is all the more justified in our context.

In our cohort, the orchiectomy rate was 44.44 % and 80.55 % respectively when the surgical management delay was less than 2 hours and more than 2 hours. However, this difference was not statistically significant (*p* = 0.06). It should be pointed out that the longer the management delay, the longer the ischemia lasts and the higher the risk of necrosis. It is therefore indispensable to avoid any factor that may lengthen the surgical management delay. It is important to avoid delay in surgical exploration because symptoms duration is already long. A duration of the symptoms more than 6 hours was a predictive factor for orchiectomy in our cohort on univariate analysis (*p* = 0.009). However, the duration of symptoms was no longer significant in multivariate analysis. This finding is contrary to what most authors report in the literature [5, 8, 11]. The delay in diagnosis when the patient arrives at the primary health-care centre is the determining factor in our context. This issue engage the responsibility of medical staff [5]. For some authors, in addition to symptoms duration, the severity of rotation is the main factor. Dias et al. [5] noted that the number of turns was significantly associated with a high orchiectomy rate, regardless of symptoms duration. Williamson [1] reported a case of orchiectomy after only 4 hours’ ischemia, and 2 viable testicles after 25 days of symptoms. He concluded that there is no absolute time beyond which one can assume that infarction is inevitable. Also Bentley et al. [16] asserted that testis perfusion can be maintained for a prolonged period in the presence of testicular torsion due to anatomical variability.

The patients’age was not a predictive factor of orchiectomy in our series (*p* = 0.69). There is a controversy concerning the role of age as a predictor of orchiectomy. Mansbach et al. [9], in a series of 436 patients under 25, found out a statistically significant correlation between the patient’s age and the occurrence of orchiectomy (*p* = 0.003). The more age increases, the more the risk of orchiectomy does. Also, for Ramachandra et al. [11], Zhao et al. [17] and Cost et al. [18], young age was significantly correlated with the risk of orchiectomy. The younger children’s inability to express scrotal pain might result in diagnosis and management delay.

## Conclusions

The orchiectomy rate was very high in our cohort. Only previous medical management was significant on multivariate analysis. This shows that delay in diagnosis is the determining factor in testicular loss. The duration of symptoms and the performance of scrotal ultrasound were just significant on univariate analysis. All of these factors contribute to a significant increase of the risk of orchiectomy. These factors are modifiable. A sensitization of all the actors is therefore necessary to reduce the orchiectomy rate. It would also be relevant to conduct a study on the fate of testicles that benefited from orchidopexy. What is the impact on testicular volume and on the spermogram?

## Data Availability

The datasets used and analysed during the current study are available from the corresponding author on reasonable request.

## Abbreviations

SCT: Spermatic cord torsion
USA: United States of America
IQR: Interquartile Range
